# Orphenadrinium picrate picric acid

**DOI:** 10.1107/S1600536810006379

**Published:** 2010-02-24

**Authors:** Hoong-Kun Fun, Madhukar Hemamalini, B. P. Siddaraju, H. S. Yathirajan, B. Narayana

**Affiliations:** a X-ray Crystallography Unit, School of Physics, Universiti Sains Malaysia, 11800 USM, Penang, Malaysia; b Department of Chemistry, V. V. Puram College of Science, Bangalore 560 004, India; c Deapartment of Studies in Chemistry, University of Mysore, Manasagangotri, Mysore 570 006, India; d Department of Studies in Chemistry, Mangalore University, Mangalagangotri 574 199, India

## Abstract

The asymmetric unit of the title compound *N*,*N*-dimethyl-2-[(2-methyl­phen­yl)phenyl­meth­oxy]ethanaminium picrate picric acid, C_18_H_24_NO^+^·C_6_H_2_N_3_O_7_^−^·C_6_H_3_N_3_O_7_, contains one orphenadrinium cation, one picrate anion and one picric acid mol­ecule. In the orphenadrine cation, the two aromatic rings form a dihedral angle of 70.30 (7)°. There is an intra­molecular O—H⋯O hydrogen bond in the picric acid mol­ecule, which generates an *S*(6) ring motif. In the crystal structure, the orphenadrine cations, picrate anions and picric acid mol­ecules are connected by strong inter­molecular N—H⋯O hydrogen bonds, π⋯π inter­actions between the benzene rings of cations and anions [centroid–centroid distance = 3.5603 (9) Å] and weak C—H⋯O hydrogen bonds, forming a three-dimensional network.

## Related literature

For the efficiency of the anti­cholinergic drug orphenadrine (systematic name *N*,*N*-dimethyl-2-[(2-methyl­phen­yl)phenyl­meth­oxy]ethanamine), see: Hunskaar & Donnel (1991 ▶). For related structures, see: Glaser *et al.* (1992 ▶); Yathirajan *et al.* (2007 ▶). For details of hydrogen bonding, see: Jeffrey & Saenger (1991 ▶); Jeffrey (1997 ▶); Scheiner (1997 ▶). For hydrogen-bond motifs, see: Bernstein *et al.* (1995 ▶). For the stability of the temperature controller used in the data collection, see: Cosier & Glazer (1986 ▶).
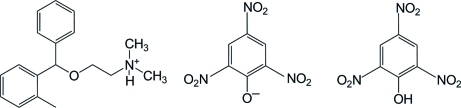


## Experimental

### 

#### Crystal data

C_18_H_24_NO^+^·C_6_H_2_N_3_O_7_^−^·C_6_H_3_N_3_O_7_
*M* *_r_* = 727.60
Monoclinic, 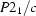

*a* = 11.1914 (9) Å
*b* = 12.4481 (10) Å
*c* = 22.6127 (19) Å
β = 93.601 (1)°
*V* = 3144.0 (4) Å^3^
*Z* = 4
Mo *K*α radiation
μ = 0.13 mm^−1^
*T* = 100 K
0.34 × 0.25 × 0.18 mm

#### Data collection

Bruker APEX DUO CCD area-detector diffractometer
Absorption correction: multi-scan (*SADABS*; Bruker, 2009 ▶) *T*_min_ = 0.959, *T*_max_ = 0.978
34841 measured reflections
9224 independent reflections
6851 reflections with *I* > 2s(*I*)
*R*_int_ = 0.039

#### Refinement

*R*[*F* ^2^ > 2σ(*F* ^2^)] = 0.044
*wR*(*F* ^2^) = 0.127
*S* = 1.02
9224 reflections
480 parameters
H atoms treated by a mixture of independent and constrained refinement
Δρ_max_ = 0.56 e Å^−3^
Δρ_min_ = −0.47 e Å^−3^

### 

Data collection: *APEX2* (Bruker, 2009 ▶); cell refinement: *SAINT* (Bruker, 2009 ▶); data reduction: *SAINT*; program(s) used to solve structure: *SHELXTL* (Sheldrick, 2008 ▶); program(s) used to refine structure: *SHELXTL*; molecular graphics: *SHELXTL*; software used to prepare material for publication: *SHELXTL* and *PLATON* (Spek, 2009 ▶).

## Supplementary Material

Crystal structure: contains datablocks global, I. DOI: 10.1107/S1600536810006379/ci5037sup1.cif

Structure factors: contains datablocks I. DOI: 10.1107/S1600536810006379/ci5037Isup2.hkl

Additional supplementary materials:  crystallographic information; 3D view; checkCIF report

## Figures and Tables

**Table 1:** Hydrogen-bond geometry (Å, °)

*D*—H⋯*A*	*D*—H	H⋯*A*	*D*⋯*A*	*D*—H⋯*A*
N4—H1*N*4⋯O1*A*	0.90 (2)	1.88 (2)	2.6860 (15)	149 (2)
N4—H1*N*4⋯O2*A*	0.90 (2)	2.32 (2)	2.9718 (16)	129 (2)
O1*B*—H1*OB*⋯O2*B*	0.99 (3)	1.66 (3)	2.5412 (15)	146 (3)
C5*B*—H5*BA*⋯O3*A*^i^	0.93	2.41	3.2833 (19)	156
C9—H9*A*⋯O6*B*^ii^	0.93	2.59	3.4884 (19)	163
C20—H20*B*⋯O4*A*^iii^	0.97	2.47	3.2841 (17)	141
C23—H23*C*⋯O7*B*^iv^	0.96	2.58	3.4901 (18)	158

Symmetry codes: (i) 
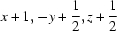
; (ii) 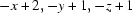; (iii) 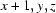; (iv) 
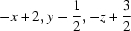
.

**Fig. 1.**
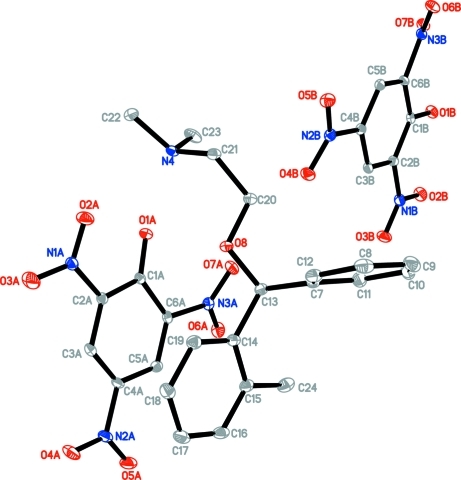
The asymmetric unit of the title compound, showing 30% probability displacement ellipsoids and the atom-numbering scheme. H atoms have been omitted for clarity.

**Fig. 2.**
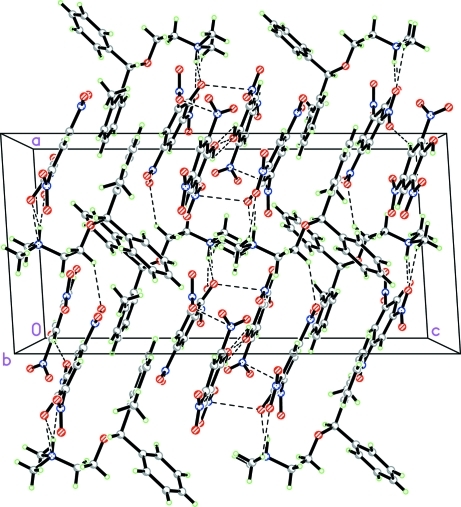
The crystal packing of the title compound, showing hydrogen-bonded (dashed lines) network.

## supplementary crystallographic information

### Comment

Orphenadrine (systematic IUPAC name: N,N-dimethyl-2-[(2-methylphenyl) phenyl-methoxy]ethanamine) is an anticholinergic drug of the ethanolamine antihistamine class with prominent CNS and peripheral actions used to treat painful muscle spasm and other symptoms and conditions as well as some aspects of Parkinson's disease. It is closely related to diphenhydramine and therefore related to other drugs used for Parkinson's like benztropine and trihexyphenidyl and is also structurally related to nefopam, a centrally-acting yet non-opioid analgesic. Clinical and pharmacological review of the efficacy of orphenadrine and its combination with paracetamol has been described (Hunskaar & Donnel, 1991). The solid-state structure of orphenadrine hydrochloride and conformational comparisons with diphenhydramine hydrochloride and nefopam hydrochloride was reported (Glaser *et al.*,  1992). The present work reports the crystal structure of the title compound which was obtained by the interaction between orphenadrine hydrochloride and 2,4,6-trinitrophenol in aqueous medium.

The asymmetric unit of the title compound (Fig. 1), contains a protonated orphenadrine cation, a picrate anion and a picric acid. In the orphenadrine cation, the two aromatic rings (C7–C12 and C14–C19) form a dihedral angle of 70.30 (7)°. The phenol and phenolate O atoms are bent slightly away from the plane of the attached benzene rings, with O1A—C1A—C2A—C3A and O1B—C1B—C2B—C3B torsion angles of 168.56 (13)° and -177.10 (13)°, respectively. The mean planes of the p-NO_2_ oxygen atoms (O4A/N2A/O5A and O4B/N2B/O5B) of picrate anion and the picric acid molecule are twisted by 4.26 (7)° and 10.12 (8)°, respectively, from the mean plane of the attached benzene rings.

In the crystal structure (Fig. 2), the  picrate anion interacts with the protonated cations through bifurcated N4—H1N4···O1A and N4—H1N4···O2A hydrogen bonds, forming R_1_^2^(6) ring motifs. This type of hydrogen bonds was also observed in the crystal structure of chloropromazinium picrate (Yathirajan *et al.*, 2007). There is an intramolecular O—H···O hydrogen bond in the picric acid molecule, which generates an S(6) ring motif. The crystal structure is further stabilized by π···π interaction between the benzene ring of the cation and anion [centroid-to-centroid distance = 3.5603 (9) Å] and several weak C—H···O hydrogen bonds (Table 1). A short O6A···N3B(-1+x, 3/2-y, -1/2+z) contact of 2.8301 (16) Å is also observed.

### Experimental

Orphenadrine hydrochloride (3.05 g, 0.01 mol) was dissolved in 25 ml of water and picric acid (2.4 g, 0.01 mol) was also dissolved in 25 ml of water. Both solutions were mixed and stirred in a beaker at room temperature for 1 h. The mixture was kept aside for 2 d at room temperature. The formed product was filtered and dried in vaccum desiccator over phosphorous pentoxide. The product was recrystallized from methanol by slow evaporation (m.p. 403-405 K).

### Refinement

Atoms H1N4 and H1O3 were located in a difference Fourier map and refined freely. The remaining H atoms were positioned geometrically [C–H = 0.93–0.98 Å] and were refined using a riding model, with *U*_iso_(H) = 1.2 or 1.5 *U*_eq_(C). A rotating group model was applied to the methyl groups.

### Crystal data

**Table:**

C_18_H_24_NO^+^·C_6_H_2_N_3_O_7_^−^·C_6_H_3_N_3_O_7_	*F*(000) = 1512
*M**_r_* = 727.60	*D*_x_ = 1.537 Mg m^−^^3^
Monoclinic, *P*2_1_/*c*	Mo *K*α radiation, λ = 0.71073 Å
Hall symbol: -P 2ybc	Cell parameters from 8774 reflections
*a* = 11.1914 (9) Å	θ = 2.4–30.0°
*b* = 12.4481 (10) Å	µ = 0.13 mm^−^^1^
*c* = 22.6127 (19) Å	*T* = 100 K
β = 93.601 (1)°	Block, yellow
*V* = 3144.0 (4) Å^3^	0.34 × 0.25 × 0.18 mm
*Z* = 4	

### Data collection

**Table:**

Bruker APEX DUO CCD area-detector diffractometer	9224 independent reflections
Radiation source: fine-focus sealed tube	6851 reflections with *I* > 2s(*I*)
graphite	*R*_int_ = 0.039
φ and ω scans	θ_max_ = 30.2°, θ_min_ = 1.8°
Absorption correction: multi-scan (*SADABS*; Bruker, 2009)	*h* = −15→15
*T*_min_ = 0.959, *T*_max_ = 0.978	*k* = −17→16
34841 measured reflections	*l* = −30→31

### Refinement

**Table:**

Refinement on *F*^2^	Primary atom site location: structure-invariant direct methods
Least-squares matrix: full	Secondary atom site location: difference Fourier map
*R*[*F*^2^ > 2σ(*F*^2^)] = 0.044	Hydrogen site location: inferred from neighbouring sites
*wR*(*F*^2^) = 0.127	H atoms treated by a mixture of independent and constrained refinement
*S* = 1.02	*w* = 1/[σ^2^(*F*_o_^2^) + (0.0621*P*)^2^ + 1.1401*P*] where *P* = (*F*_o_^2^ + 2*F*_c_^2^)/3
9224 reflections	(Δ/σ)_max_ = 0.001
480 parameters	Δρ_max_ = 0.56 e Å^−^^3^
0 restraints	Δρ_min_ = −0.47 e Å^−^^3^

### Special details

**Table:**

Experimental. The crystal was placed in the cold stream of an Oxford Cryosystems Cobra open-flow nitrogen cryostat (Cosier & Glazer, 1986) operating at 100.0 (1) K.
Geometry. All esds (except the esd in the dihedral angle between two l.s. planes) are estimated using the full covariance matrix. The cell esds are taken into account individually in the estimation of esds in distances, angles and torsion angles; correlations between esds in cell parameters are only used when they are defined by crystal symmetry. An approximate (isotropic) treatment of cell esds is used for estimating esds involving l.s. planes.
Refinement. Refinement of F^2^ against ALL reflections. The weighted R-factor wR and goodness of fit S are based on F^2^, conventional R-factors R are based on F, with F set to zero for negative F^2^. The threshold expression of F^2^ > 2sigma(F^2^) is used only for calculating R-factors(gt) etc. and is not relevant to the choice of reflections for refinement. R-factors based on F^2^ are statistically about twice as large as those based on F, and R- factors based on ALL data will be even larger.

### Fractional atomic coordinates and isotropic or equivalent isotropic displacement parameters (Å^2^)

**Table:**

	*x*	*y*	*z*	*U*_iso_*/*U*_eq_	
O1A	0.29799 (8)	0.35411 (8)	0.45233 (4)	0.0182 (2)	
O2A	0.26460 (9)	0.14380 (9)	0.43358 (6)	0.0318 (3)	
O3A	0.08488 (11)	0.09425 (10)	0.41005 (8)	0.0455 (4)	
O4A	−0.19368 (9)	0.32322 (10)	0.30733 (6)	0.0324 (3)	
O5A	−0.16667 (10)	0.49561 (10)	0.30775 (6)	0.0312 (3)	
O6A	0.19194 (10)	0.64682 (9)	0.40080 (5)	0.0255 (2)	
O7A	0.34962 (9)	0.54552 (9)	0.40303 (5)	0.0236 (2)	
N1A	0.16227 (10)	0.16434 (10)	0.41499 (6)	0.0193 (2)	
N2A	−0.13373 (10)	0.40319 (11)	0.31998 (6)	0.0229 (3)	
N3A	0.24039 (10)	0.55773 (10)	0.40000 (5)	0.0177 (2)	
C1A	0.20748 (11)	0.36151 (11)	0.41721 (6)	0.0149 (3)	
C2A	0.12853 (11)	0.27347 (11)	0.39928 (6)	0.0156 (3)	
C3A	0.01861 (11)	0.28696 (12)	0.36858 (6)	0.0179 (3)	
H3AA	−0.0302	0.2280	0.3596	0.021*	
C4A	−0.01768 (11)	0.38852 (12)	0.35144 (6)	0.0180 (3)	
C5A	0.05516 (12)	0.47732 (12)	0.36265 (6)	0.0177 (3)	
H5AA	0.0303	0.5453	0.3500	0.021*	
C6A	0.16441 (11)	0.46326 (11)	0.39269 (6)	0.0156 (3)	
O1B	0.97430 (9)	0.90711 (9)	0.97661 (5)	0.0211 (2)	
O2B	0.77967 (10)	0.99530 (9)	0.93846 (5)	0.0260 (2)	
O3B	0.63236 (9)	0.90050 (9)	0.90035 (6)	0.0278 (3)	
O4B	0.65060 (9)	0.52065 (9)	0.89499 (5)	0.0262 (2)	
O5B	0.82135 (10)	0.43835 (9)	0.90729 (5)	0.0248 (2)	
O6B	1.17444 (9)	0.64338 (9)	0.97116 (5)	0.0243 (2)	
O7B	1.14073 (9)	0.78048 (9)	1.02692 (5)	0.0217 (2)	
N1B	0.73404 (10)	0.90814 (10)	0.92275 (6)	0.0201 (2)	
N2B	0.75924 (10)	0.51912 (10)	0.90699 (5)	0.0181 (2)	
N3B	1.10999 (10)	0.71454 (10)	0.98881 (5)	0.0174 (2)	
C1B	0.92362 (11)	0.81618 (11)	0.95787 (6)	0.0153 (3)	
C2B	0.80552 (11)	0.81061 (11)	0.93132 (6)	0.0164 (3)	
C3B	0.75193 (11)	0.71467 (12)	0.91434 (6)	0.0168 (3)	
H3BA	0.6737	0.7129	0.8979	0.020*	
C4B	0.81729 (11)	0.62166 (11)	0.92236 (6)	0.0159 (3)	
C5B	0.93580 (11)	0.62156 (11)	0.94473 (6)	0.0161 (3)	
H5BA	0.9799	0.5582	0.9475	0.019*	
C6B	0.98593 (11)	0.71820 (11)	0.96275 (6)	0.0153 (3)	
O8	0.41316 (8)	0.30752 (8)	0.32088 (4)	0.0186 (2)	
N4	0.50481 (10)	0.24548 (10)	0.44887 (5)	0.0170 (2)	
C7	0.49369 (13)	0.30347 (13)	0.19733 (7)	0.0230 (3)	
H7A	0.4506	0.2408	0.2031	0.028*	
C8	0.58017 (14)	0.30557 (16)	0.15569 (7)	0.0300 (4)	
H8A	0.5954	0.2441	0.1340	0.036*	
C9	0.64370 (14)	0.39915 (17)	0.14640 (7)	0.0341 (4)	
H9A	0.7009	0.4006	0.1183	0.041*	
C10	0.62187 (14)	0.48991 (17)	0.17894 (8)	0.0325 (4)	
H10A	0.6643	0.5527	0.1727	0.039*	
C11	0.53631 (13)	0.48776 (14)	0.22113 (7)	0.0247 (3)	
H11A	0.5225	0.5490	0.2432	0.030*	
C12	0.47145 (12)	0.39479 (12)	0.23045 (6)	0.0186 (3)	
C13	0.38138 (11)	0.39039 (12)	0.27850 (6)	0.0174 (3)	
H13A	0.3829	0.4596	0.2992	0.021*	
C14	0.25466 (12)	0.37030 (12)	0.25284 (6)	0.0187 (3)	
C15	0.18191 (13)	0.45666 (13)	0.23244 (6)	0.0217 (3)	
C16	0.06643 (13)	0.43435 (15)	0.20804 (7)	0.0263 (3)	
H16A	0.0179	0.4909	0.1944	0.032*	
C17	0.02249 (13)	0.33060 (16)	0.20362 (7)	0.0286 (4)	
H17A	−0.0545	0.3178	0.1873	0.034*	
C18	0.09432 (13)	0.24604 (15)	0.22372 (7)	0.0262 (3)	
H18A	0.0657	0.1760	0.2209	0.031*	
C19	0.20997 (13)	0.26597 (13)	0.24823 (7)	0.0224 (3)	
H19A	0.2578	0.2088	0.2617	0.027*	
C20	0.52997 (12)	0.32655 (13)	0.34831 (7)	0.0230 (3)	
H20A	0.5310	0.3945	0.3694	0.028*	
H20B	0.5881	0.3304	0.3183	0.028*	
C21	0.56231 (12)	0.23669 (12)	0.39088 (6)	0.0195 (3)	
H21A	0.5389	0.1690	0.3723	0.023*	
H21B	0.6486	0.2355	0.3985	0.023*	
C22	0.51695 (13)	0.14244 (14)	0.48196 (7)	0.0270 (3)	
H22A	0.4817	0.0855	0.4582	0.040*	
H22B	0.4768	0.1480	0.5181	0.040*	
H22C	0.6002	0.1273	0.4910	0.040*	
C23	0.55619 (13)	0.33515 (14)	0.48613 (8)	0.0274 (3)	
H23A	0.5169	0.3381	0.5226	0.041*	
H23B	0.5445	0.4018	0.4653	0.041*	
H23C	0.6403	0.3232	0.4945	0.041*	
C24	0.22435 (15)	0.57185 (14)	0.23662 (8)	0.0297 (4)	
H24A	0.1601	0.6189	0.2235	0.045*	
H24B	0.2904	0.5815	0.2121	0.045*	
H24C	0.2495	0.5882	0.2770	0.045*	
H1N4	0.4270 (16)	0.2605 (15)	0.4420 (8)	0.022 (4)*	
H1OB	0.914 (3)	0.964 (2)	0.9690 (13)	0.077 (9)*	

### Atomic displacement parameters (Å^2^)

**Table:**

	*U*^11^	*U*^22^	*U*^33^	*U*^12^	*U*^13^	*U*^23^
O1A	0.0146 (4)	0.0210 (5)	0.0186 (5)	0.0008 (4)	−0.0020 (4)	−0.0019 (4)
O2A	0.0215 (5)	0.0204 (6)	0.0521 (8)	0.0028 (4)	−0.0079 (5)	0.0023 (5)
O3A	0.0318 (6)	0.0223 (7)	0.0799 (11)	−0.0109 (5)	−0.0165 (7)	0.0081 (7)
O4A	0.0176 (5)	0.0352 (7)	0.0434 (7)	−0.0039 (5)	−0.0069 (5)	−0.0041 (6)
O5A	0.0226 (5)	0.0316 (7)	0.0383 (7)	0.0064 (5)	−0.0072 (5)	0.0054 (5)
O6A	0.0285 (5)	0.0156 (5)	0.0331 (6)	0.0009 (4)	0.0071 (5)	−0.0015 (4)
O7A	0.0166 (4)	0.0241 (6)	0.0300 (6)	−0.0042 (4)	0.0002 (4)	−0.0033 (5)
N1A	0.0167 (5)	0.0174 (6)	0.0238 (6)	−0.0011 (4)	0.0005 (4)	0.0002 (5)
N2A	0.0144 (5)	0.0309 (7)	0.0231 (6)	0.0014 (5)	−0.0012 (4)	−0.0009 (5)
N3A	0.0196 (5)	0.0179 (6)	0.0157 (6)	−0.0017 (4)	0.0026 (4)	−0.0022 (5)
C1A	0.0130 (5)	0.0173 (7)	0.0147 (6)	0.0000 (5)	0.0034 (4)	−0.0022 (5)
C2A	0.0144 (5)	0.0154 (7)	0.0171 (6)	0.0007 (5)	0.0021 (5)	−0.0002 (5)
C3A	0.0137 (5)	0.0208 (7)	0.0194 (7)	−0.0023 (5)	0.0028 (5)	−0.0026 (5)
C4A	0.0116 (5)	0.0240 (7)	0.0182 (7)	0.0005 (5)	−0.0012 (5)	−0.0004 (5)
C5A	0.0171 (6)	0.0193 (7)	0.0169 (6)	0.0033 (5)	0.0024 (5)	0.0010 (5)
C6A	0.0157 (5)	0.0157 (6)	0.0158 (6)	−0.0019 (5)	0.0033 (5)	−0.0019 (5)
O1B	0.0189 (4)	0.0162 (5)	0.0282 (6)	−0.0028 (4)	0.0020 (4)	−0.0043 (4)
O2B	0.0260 (5)	0.0148 (5)	0.0375 (7)	−0.0002 (4)	0.0045 (5)	−0.0014 (5)
O3B	0.0183 (5)	0.0240 (6)	0.0407 (7)	0.0041 (4)	−0.0022 (4)	0.0031 (5)
O4B	0.0192 (5)	0.0237 (6)	0.0346 (6)	−0.0067 (4)	−0.0071 (4)	0.0016 (5)
O5B	0.0277 (5)	0.0157 (5)	0.0304 (6)	0.0002 (4)	−0.0020 (4)	−0.0001 (4)
O6B	0.0175 (4)	0.0266 (6)	0.0289 (6)	0.0042 (4)	0.0022 (4)	−0.0047 (5)
O7B	0.0193 (4)	0.0233 (6)	0.0221 (5)	−0.0036 (4)	−0.0025 (4)	−0.0050 (4)
N1B	0.0188 (5)	0.0164 (6)	0.0254 (6)	0.0012 (4)	0.0046 (5)	0.0016 (5)
N2B	0.0203 (5)	0.0157 (6)	0.0179 (6)	−0.0031 (4)	−0.0013 (4)	0.0007 (5)
N3B	0.0149 (5)	0.0198 (6)	0.0174 (6)	−0.0019 (4)	0.0011 (4)	0.0002 (5)
C1B	0.0155 (5)	0.0156 (6)	0.0153 (6)	−0.0026 (5)	0.0043 (5)	−0.0011 (5)
C2B	0.0159 (5)	0.0153 (6)	0.0183 (6)	0.0015 (5)	0.0034 (5)	0.0005 (5)
C3B	0.0147 (5)	0.0190 (7)	0.0168 (6)	−0.0020 (5)	0.0015 (5)	0.0010 (5)
C4B	0.0181 (6)	0.0146 (6)	0.0149 (6)	−0.0033 (5)	0.0010 (5)	−0.0005 (5)
C5B	0.0171 (6)	0.0161 (6)	0.0151 (6)	0.0001 (5)	0.0014 (5)	0.0003 (5)
C6B	0.0126 (5)	0.0172 (6)	0.0160 (6)	−0.0015 (5)	0.0010 (4)	−0.0002 (5)
O8	0.0160 (4)	0.0225 (5)	0.0170 (5)	0.0011 (4)	−0.0023 (4)	0.0033 (4)
N4	0.0119 (5)	0.0211 (6)	0.0175 (6)	0.0006 (4)	−0.0019 (4)	−0.0009 (5)
C7	0.0231 (6)	0.0270 (8)	0.0187 (7)	0.0070 (6)	−0.0006 (5)	−0.0007 (6)
C8	0.0263 (7)	0.0432 (10)	0.0202 (7)	0.0137 (7)	−0.0004 (6)	−0.0055 (7)
C9	0.0198 (6)	0.0622 (13)	0.0205 (8)	0.0027 (7)	0.0033 (6)	−0.0003 (8)
C10	0.0247 (7)	0.0484 (11)	0.0242 (8)	−0.0097 (7)	0.0017 (6)	0.0040 (8)
C11	0.0236 (6)	0.0303 (8)	0.0200 (7)	−0.0031 (6)	0.0009 (6)	−0.0009 (6)
C12	0.0161 (5)	0.0246 (7)	0.0149 (6)	0.0044 (5)	−0.0009 (5)	0.0012 (5)
C13	0.0177 (6)	0.0191 (7)	0.0152 (6)	0.0031 (5)	0.0007 (5)	0.0008 (5)
C14	0.0170 (6)	0.0251 (7)	0.0140 (6)	0.0042 (5)	0.0024 (5)	−0.0003 (5)
C15	0.0211 (6)	0.0290 (8)	0.0151 (6)	0.0075 (6)	0.0031 (5)	0.0019 (6)
C16	0.0199 (6)	0.0407 (10)	0.0184 (7)	0.0111 (6)	0.0018 (5)	0.0033 (6)
C17	0.0172 (6)	0.0504 (11)	0.0180 (7)	0.0016 (6)	0.0001 (5)	−0.0016 (7)
C18	0.0230 (7)	0.0350 (9)	0.0207 (7)	−0.0049 (6)	0.0019 (6)	−0.0037 (7)
C19	0.0217 (6)	0.0259 (8)	0.0195 (7)	0.0027 (6)	0.0006 (5)	−0.0008 (6)
C20	0.0170 (6)	0.0291 (8)	0.0224 (7)	−0.0016 (6)	−0.0038 (5)	0.0061 (6)
C21	0.0173 (6)	0.0238 (7)	0.0172 (7)	0.0054 (5)	−0.0001 (5)	0.0000 (6)
C22	0.0224 (7)	0.0326 (9)	0.0259 (8)	0.0047 (6)	0.0007 (6)	0.0110 (7)
C23	0.0172 (6)	0.0350 (9)	0.0294 (8)	−0.0003 (6)	−0.0026 (6)	−0.0137 (7)
C24	0.0345 (8)	0.0284 (9)	0.0261 (8)	0.0094 (7)	0.0003 (6)	0.0032 (7)

### Geometric parameters (Å, °)

**Table:**

O1A—C1A	1.2507 (16)	N4—C21	1.5004 (18)
O2A—N1A	1.2219 (15)	N4—H1N4	0.894 (18)
O3A—N1A	1.2296 (16)	C7—C12	1.392 (2)
O4A—N2A	1.2243 (18)	C7—C8	1.392 (2)
O5A—N2A	1.2342 (18)	C7—H7A	0.93
O6A—N3A	1.2351 (16)	C8—C9	1.388 (3)
O7A—N3A	1.2295 (15)	C8—H8A	0.93
N1A—C2A	1.4482 (18)	C9—C10	1.378 (3)
N2A—C4A	1.4527 (17)	C9—H9A	0.93
N3A—C6A	1.4545 (18)	C10—C11	1.394 (2)
C1A—C2A	1.4495 (19)	C10—H10A	0.93
C1A—C6A	1.4528 (19)	C11—C12	1.389 (2)
C2A—C3A	1.3842 (18)	C11—H11A	0.93
C3A—C4A	1.376 (2)	C12—C13	1.5288 (19)
C3A—H3AA	0.93	C13—C14	1.5188 (19)
C4A—C5A	1.387 (2)	C13—H13A	0.98
C5A—C6A	1.3720 (19)	C14—C19	1.393 (2)
C5A—H5AA	0.93	C14—C15	1.409 (2)
O1B—C1B	1.3237 (17)	C15—C16	1.401 (2)
O1B—H1OB	0.99 (3)	C15—C24	1.512 (2)
O2B—N1B	1.2416 (17)	C16—C17	1.383 (3)
O3B—N1B	1.2200 (16)	C16—H16A	0.93
O4B—N2B	1.2290 (15)	C17—C18	1.384 (2)
O5B—N2B	1.2222 (16)	C17—H17A	0.93
O6B—N3B	1.2249 (15)	C18—C19	1.398 (2)
O7B—N3B	1.2238 (16)	C18—H18A	0.93
N1B—C2B	1.4601 (18)	C19—H19A	0.93
N2B—C4B	1.4642 (18)	C20—C21	1.505 (2)
N3B—C6B	1.4745 (16)	C20—H20A	0.97
C1B—C6B	1.4059 (19)	C20—H20B	0.97
C1B—C2B	1.4186 (18)	C21—H21A	0.97
C2B—C3B	1.380 (2)	C21—H21B	0.97
C3B—C4B	1.3754 (19)	C22—H22A	0.96
C3B—H3BA	0.93	C22—H22B	0.96
C4B—C5B	1.3895 (18)	C22—H22C	0.96
C5B—C6B	1.3782 (19)	C23—H23A	0.96
C5B—H5BA	0.93	C23—H23B	0.96
O8—C20	1.4311 (16)	C23—H23C	0.96
O8—C13	1.4375 (17)	C24—H24A	0.96
N4—C22	1.487 (2)	C24—H24B	0.96
N4—C23	1.4919 (19)	C24—H24C	0.96
			
O2A—N1A—O3A	121.49 (13)	C7—C8—H8A	119.9
O2A—N1A—C2A	120.33 (12)	C10—C9—C8	119.84 (15)
O3A—N1A—C2A	118.17 (12)	C10—C9—H9A	120.1
O4A—N2A—O5A	123.64 (12)	C8—C9—H9A	120.1
O4A—N2A—C4A	118.18 (13)	C9—C10—C11	120.14 (17)
O5A—N2A—C4A	118.18 (13)	C9—C10—H10A	119.9
O7A—N3A—O6A	123.09 (12)	C11—C10—H10A	119.9
O7A—N3A—C6A	118.59 (12)	C12—C11—C10	120.51 (16)
O6A—N3A—C6A	118.27 (11)	C12—C11—H11A	119.7
O1A—C1A—C2A	125.25 (13)	C10—C11—H11A	119.7
O1A—C1A—C6A	122.86 (12)	C11—C12—C7	119.07 (13)
C2A—C1A—C6A	111.75 (11)	C11—C12—C13	120.78 (13)
C3A—C2A—N1A	116.53 (12)	C7—C12—C13	120.08 (13)
C3A—C2A—C1A	123.74 (13)	O8—C13—C14	108.81 (11)
N1A—C2A—C1A	119.71 (11)	O8—C13—C12	110.66 (11)
C4A—C3A—C2A	119.34 (13)	C14—C13—C12	112.08 (11)
C4A—C3A—H3AA	120.3	O8—C13—H13A	108.4
C2A—C3A—H3AA	120.3	C14—C13—H13A	108.4
C3A—C4A—C5A	121.39 (12)	C12—C13—H13A	108.4
C3A—C4A—N2A	119.50 (13)	C19—C14—C15	119.31 (13)
C5A—C4A—N2A	119.10 (13)	C19—C14—C13	120.28 (13)
C6A—C5A—C4A	118.94 (13)	C15—C14—C13	120.40 (13)
C6A—C5A—H5AA	120.5	C16—C15—C14	118.53 (15)
C4A—C5A—H5AA	120.5	C16—C15—C24	119.42 (14)
C5A—C6A—C1A	124.35 (12)	C14—C15—C24	122.04 (13)
C5A—C6A—N3A	116.71 (12)	C17—C16—C15	121.91 (15)
C1A—C6A—N3A	118.94 (11)	C17—C16—H16A	119.0
C1B—O1B—H1OB	106.4 (17)	C15—C16—H16A	119.0
O3B—N1B—O2B	122.90 (12)	C16—C17—C18	119.33 (14)
O3B—N1B—C2B	118.64 (12)	C16—C17—H17A	120.3
O2B—N1B—C2B	118.47 (12)	C18—C17—H17A	120.3
O5B—N2B—O4B	124.64 (12)	C17—C18—C19	119.97 (16)
O5B—N2B—C4B	118.17 (11)	C17—C18—H18A	120.0
O4B—N2B—C4B	117.19 (12)	C19—C18—H18A	120.0
O7B—N3B—O6B	124.70 (12)	C14—C19—C18	120.94 (15)
O7B—N3B—C6B	118.47 (11)	C14—C19—H19A	119.5
O6B—N3B—C6B	116.81 (12)	C18—C19—H19A	119.5
O1B—C1B—C6B	121.08 (12)	O8—C20—C21	109.30 (12)
O1B—C1B—C2B	123.12 (12)	O8—C20—H20A	109.8
C6B—C1B—C2B	115.80 (12)	C21—C20—H20A	109.8
C3B—C2B—C1B	122.49 (13)	O8—C20—H20B	109.8
C3B—C2B—N1B	117.24 (12)	C21—C20—H20B	109.8
C1B—C2B—N1B	120.24 (12)	H20A—C20—H20B	108.3
C4B—C3B—C2B	118.26 (12)	N4—C21—C20	113.93 (12)
C4B—C3B—H3BA	120.9	N4—C21—H21A	108.8
C2B—C3B—H3BA	120.9	C20—C21—H21A	108.8
C3B—C4B—C5B	122.44 (13)	N4—C21—H21B	108.8
C3B—C4B—N2B	118.56 (12)	C20—C21—H21B	108.8
C5B—C4B—N2B	119.00 (12)	H21A—C21—H21B	107.7
C6B—C5B—C4B	117.94 (13)	N4—C22—H22A	109.5
C6B—C5B—H5BA	121.0	N4—C22—H22B	109.5
C4B—C5B—H5BA	121.0	H22A—C22—H22B	109.5
C5B—C6B—C1B	122.91 (12)	N4—C22—H22C	109.5
C5B—C6B—N3B	116.54 (12)	H22A—C22—H22C	109.5
C1B—C6B—N3B	120.54 (12)	H22B—C22—H22C	109.5
C20—O8—C13	110.56 (11)	N4—C23—H23A	109.5
C22—N4—C23	109.96 (12)	N4—C23—H23B	109.5
C22—N4—C21	110.26 (12)	H23A—C23—H23B	109.5
C23—N4—C21	112.14 (12)	N4—C23—H23C	109.5
C22—N4—H1N4	108.9 (12)	H23A—C23—H23C	109.5
C23—N4—H1N4	106.1 (12)	H23B—C23—H23C	109.5
C21—N4—H1N4	109.3 (12)	C15—C24—H24A	109.5
C12—C7—C8	120.25 (16)	C15—C24—H24B	109.5
C12—C7—H7A	119.9	H24A—C24—H24B	109.5
C8—C7—H7A	119.9	C15—C24—H24C	109.5
C9—C8—C7	120.18 (16)	H24A—C24—H24C	109.5
C9—C8—H8A	119.9	H24B—C24—H24C	109.5
			
O2A—N1A—C2A—C3A	168.43 (13)	N2B—C4B—C5B—C6B	−175.54 (12)
O3A—N1A—C2A—C3A	−12.8 (2)	C4B—C5B—C6B—C1B	−1.9 (2)
O2A—N1A—C2A—C1A	−13.2 (2)	C4B—C5B—C6B—N3B	177.26 (12)
O3A—N1A—C2A—C1A	165.56 (14)	O1B—C1B—C6B—C5B	178.87 (13)
O1A—C1A—C2A—C3A	168.56 (13)	C2B—C1B—C6B—C5B	−1.84 (19)
C6A—C1A—C2A—C3A	−7.27 (18)	O1B—C1B—C6B—N3B	−0.22 (19)
O1A—C1A—C2A—N1A	−9.7 (2)	C2B—C1B—C6B—N3B	179.08 (11)
C6A—C1A—C2A—N1A	174.46 (11)	O7B—N3B—C6B—C5B	−148.70 (13)
N1A—C2A—C3A—C4A	−178.86 (12)	O6B—N3B—C6B—C5B	29.92 (17)
C1A—C2A—C3A—C4A	2.8 (2)	O7B—N3B—C6B—C1B	30.44 (18)
C2A—C3A—C4A—C5A	2.0 (2)	O6B—N3B—C6B—C1B	−150.94 (13)
C2A—C3A—C4A—N2A	−179.11 (12)	C12—C7—C8—C9	−0.8 (2)
O4A—N2A—C4A—C3A	−3.2 (2)	C7—C8—C9—C10	0.6 (2)
O5A—N2A—C4A—C3A	176.42 (13)	C8—C9—C10—C11	0.1 (3)
O4A—N2A—C4A—C5A	175.66 (13)	C9—C10—C11—C12	−0.6 (2)
O5A—N2A—C4A—C5A	−4.7 (2)	C10—C11—C12—C7	0.4 (2)
C3A—C4A—C5A—C6A	−1.5 (2)	C10—C11—C12—C13	177.33 (14)
N2A—C4A—C5A—C6A	179.62 (12)	C8—C7—C12—C11	0.3 (2)
C4A—C5A—C6A—C1A	−3.9 (2)	C8—C7—C12—C13	−176.63 (13)
C4A—C5A—C6A—N3A	176.24 (12)	C20—O8—C13—C14	−177.11 (11)
O1A—C1A—C6A—C5A	−168.09 (13)	C20—O8—C13—C12	59.33 (15)
C2A—C1A—C6A—C5A	7.87 (18)	C11—C12—C13—O8	−120.56 (14)
O1A—C1A—C6A—N3A	11.76 (19)	C7—C12—C13—O8	56.29 (17)
C2A—C1A—C6A—N3A	−172.28 (11)	C11—C12—C13—C14	117.78 (15)
O7A—N3A—C6A—C5A	−150.15 (13)	C7—C12—C13—C14	−65.37 (17)
O6A—N3A—C6A—C5A	27.39 (18)	O8—C13—C14—C19	−29.84 (17)
O7A—N3A—C6A—C1A	29.99 (18)	C12—C13—C14—C19	92.88 (16)
O6A—N3A—C6A—C1A	−152.48 (12)	O8—C13—C14—C15	151.37 (13)
O1B—C1B—C2B—C3B	−177.10 (13)	C12—C13—C14—C15	−85.91 (16)
C6B—C1B—C2B—C3B	3.62 (19)	C19—C14—C15—C16	0.0 (2)
O1B—C1B—C2B—N1B	0.8 (2)	C13—C14—C15—C16	178.78 (12)
C6B—C1B—C2B—N1B	−178.46 (12)	C19—C14—C15—C24	179.42 (14)
O3B—N1B—C2B—C3B	−1.22 (19)	C13—C14—C15—C24	−1.8 (2)
O2B—N1B—C2B—C3B	178.99 (13)	C14—C15—C16—C17	0.1 (2)
O3B—N1B—C2B—C1B	−179.25 (13)	C24—C15—C16—C17	−179.37 (14)
O2B—N1B—C2B—C1B	0.97 (19)	C15—C16—C17—C18	−0.1 (2)
C1B—C2B—C3B—C4B	−1.6 (2)	C16—C17—C18—C19	0.1 (2)
N1B—C2B—C3B—C4B	−179.57 (12)	C15—C14—C19—C18	0.0 (2)
C2B—C3B—C4B—C5B	−2.4 (2)	C13—C14—C19—C18	−178.82 (13)
C2B—C3B—C4B—N2B	177.23 (12)	C17—C18—C19—C14	0.0 (2)
O5B—N2B—C4B—C3B	171.21 (13)	C13—O8—C20—C21	−178.14 (12)
O4B—N2B—C4B—C3B	−9.23 (19)	C22—N4—C21—C20	166.36 (12)
O5B—N2B—C4B—C5B	−9.11 (19)	C23—N4—C21—C20	−70.75 (15)
O4B—N2B—C4B—C5B	170.45 (12)	O8—C20—C21—N4	−78.40 (16)
C3B—C4B—C5B—C6B	4.1 (2)		

### Hydrogen-bond geometry (Å, °)

**Table:**

*D*—H···*A*	*D*—H	H···*A*	*D*···*A*	*D*—H···*A*
N4—H1N4···O1A	0.90 (2)	1.88 (2)	2.6860 (15)	149 (2)
N4—H1N4···O2A	0.90 (2)	2.32 (2)	2.9718 (16)	129 (2)
O1B—H1OB···O2B	0.99 (3)	1.66 (3)	2.5412 (15)	146 (3)
C5B—H5BA···O3A^i^	0.93	2.41	3.2833 (19)	156
C9—H9A···O6B^ii^	0.93	2.59	3.4884 (19)	163
C20—H20B···O4A^iii^	0.97	2.47	3.2841 (17)	141
C23—H23C···O7B^iv^	0.96	2.58	3.4901 (18)	158

Symmetry codes: (i) *x*+1, −*y*+1/2, *z*+1/2; (ii) −*x*+2, −*y*+1, −*z*+1; (iii) *x*+1, *y*, *z*; (iv) −*x*+2, *y*−1/2, −*z*+3/2.
